# Efficacy and safety of 2% isosorbide cream in systemic sclerosis patients with digital ulcers and Raynaud’s phenomenon

**DOI:** 10.1515/rir-2025-0006

**Published:** 2025-04-02

**Authors:** Suwassa Namvijit, Chingching Foocharoen, Siraphop Suwannaroj, Patnarin Pongkulkait, Tippawan Onchan, Ajanee Mahakkanukrauh

**Affiliations:** Department of Pharmacy, Faculty of Medicine, Khon Kaen University, Khon Kaen 40002, Thailand; Department of Medicine, Faculty of Medicine, Khon Kaen University, Khon Kaen 40002, Thailand

**Keywords:** systemic sclerosis, scleroderma, digital ulcer, Raynaud’s phenomenon, topical nitrate, vasodilators

## Abstract

**Background and Objectives:**

Raynaud’s phenomenon (RP) and digital ulcers (DUs) impact the quality of life (QoL) of systemic sclerosis (SSc) patients. Calcium channel blockers (CCBs) and phosphodiesterase-5 inhibitors (PDE-5i) have been used to improve blood flow. However, vasodilators are limited in patients with low blood pressure. We aimed to determine the eficacy and safety of 2% isosorbide dinitrate (ISDN) cream as an adjunctive for treating DUs and RP.

**Methods:**

A cohort study was conducted at the Scleroderma Clinic at Khon Kaen University’s Srinagarind Hospital in Khon Kaen, Thailand between January 2021 and December 2022. The study included adult SSc patients, who had received 2% ISDN cream for treatment of DUs or RP as adjuvant and with/or without receiving CCBs and/or PDE-5i as a background treatment for DUs and RP. Patients had to have follow-up data between 2 and 4 months after starting treatment. The median treatment duration was 2.8 months. Dosages of sustained-release nifedipine ranged from 10–80 mg and sildenafil ranged from 12.5–150 mg. Topical 2% ISDN cream was thinly applied three times a day. The treatment responses of DUs (ulcer size and pain) and RP (frequency and duration of attack) were according to patients’ self-assessment, categorized into 3 levels Improvement Stable and Worsening. Before and after the treatment period of 2% ISDN cream the Quality of life was evaluated by using the EuroQoL five dimensions (EQ-5D) assessment by attending physicians.

**Results:**

In terms of anxiety, QoL, as evaluated by the EQ-5D, significantly improved after treatment with 2% ISDN cream in patients with DUs compared to before treatment. When used as an adjunct to CCB, 2% ISDN cream resulted in stability or improvement of RP in 43.2% of patients, DUs in 41.4% of patients, and both RP and DUs in 41.0% of patients. When used as an adjunct to PDE-5i as background therapy, it resulted in stability or improvement of RP in 13.5% of patients, DUs in 13.8% of patients, and both RP and DUs in 15.4% of patients.

**Conclusions:**

Topical 2% ISDN cream may help to reduce anxiety when DUs were improved and improve overall QoL.

## Introduction

Systemic sclerosis (SSc) is an autoimmune disease in which vasculopathy is one of the major pathogenesis and results in vascular complications.^[1]^ Raynaud’s phenomenon (RP) is a common clinical feature in patients with SSc. RP represents structural abnormalities of the microvasculature and leads to symptoms of finger pain and/or paresthesia. It can result in significant tissue ischemia including the development of digital ulcers (DUs) in cases of persistent alterations of the blood flow.^[2]^

DUs are a common and debilitating ischemic manifestation in SSc, representing end-organ damage from progressive vasculopathy and serving as a biomarker of disease severity and internal organ involvement. The presence of DUs at any time is associated with DUs recurrence, gastrointestinal involvement, and increased SSc-related mortality. DUs require close monitoring by the healthcare system for meticulous wound care and suficient analgesia, as well as for the detection of potential complications, such as the development of local and/or underlying bone infections that require prompt therapy.^[3]^

Calcium channel blockers (CCBs) and phosphodiesterase-5 inhibitors (PDE-5i) are the treatment of choice for RP and DUs. The 2016 European League Against Rheumatism (EULAR) recommendations advise oral CCBs as first-line therapy for SSc-related RP and PDE-5i for SSc patients with severe RP or for those, who do not respond to CCBs. CCBs and PDE-5i showed moderate efficacy in reducing the frequency and the severity of RP in patients with SSc.^[4]^

Alternative therapy to treat failure using CCBs and PDE-5i is treatment with prostacyclin analogues, particularly iloprost, in severe RP or failing first-line CCBs treatment.^[5]^ Intravenous (IV) iloprost is reserved for severe RP and after oral treatment. Iloprost is still not used as a treatment for DUs or RP in our daily practice due to budgetary limitations. Topical nitrates, such as nitroglycerin, which is a vasodilator is another option of treatment. Nitric oxide donor isosorbide di-nitrate (ISDN) could cause vasodilation and could, thereby, improve tissue blood distribution in the affected extremity. A systematic review and meta-analysis of the effects of topical nitrates in the treatment of primary and secondary RP with respect to combined endpoint integrating parameters of digital blood flow and clinical severity showed that multiple placebo-controlled trials had assessed locally applied topical nitrate preparations in treating RP. Local topical nitrates have significant efficacy in the treatment of both primary and secondary RP.^[6]^ However, these products are not available in Thailand. Therefore, initially the pharmacist in the Scleroderma Clinic prepared our own topical nitrate cream for Scleroderma patients, who needed topical adjuvant therapy for RPs and DUs. This preparation is now produced in the hospital and is made by the Pharmacy Production Department.

Our daily practice revealed the limitation in titrating CCB and/or PDE-5i for controlling RP and/or DUs due to low blood pressure among SSc patients, which had caused some patients not to receive any vasodilators due to their side effects. As a result, we would like to define the efficacy and safety of topical 2% ISDN cream for RP and/or DUs in SSc patients receiving CCBs and/or PDE-5i for RP and/or DUs as background therapy.

## Materials and Methods

This cohort study with retrospective analysis was conducted by reviewing the data from the SSc database registry of those patients, who had received follow up in the Scleroderma Clinic at Khon Kaen University’s Srinagarind Hospital in Khon Kaen, Thailand between January 2021 and December 2022. Treatment of RP and DUs followed the 2016 EULAR guidelines, which recommend CCBs as first-line therapy and PDE-5i for patients unresponsive to CCBs. Sustained-release nifedipine (10-80 mg daily) was the primary CCB used, while sildenafil (12.5-150 mg daily) was the PDE-5i used. Combination therapy with CCBs and PDE-5i was reserved for patients with severe RP or DUs not responsive to mono-therapy. PDE-5i was used alone in cases where CCBs were contraindicated, such as in patients with low blood pressure, or in patients with pulmonary hypertension, where PDE-5i was prioritized due to its additional benefits.

Patients were trained on the proper application of 2% ISDN cream, which was applied thinly to the wrist three times daily. Treatment responses were assessed based on patient-reported outcomes for RP frequency/duration and DU size/pain, categorized as improvement, stability, or worsening.

Adult SSc patients, who had received 2% ISDN cream for treatment of DUs or RP for at least 1 month and with/or without receiving CCB and/or PDE-5i as a background treatment for DUs and RP, were included. The dosage of sustained-release nifedipine ranged from 10–80 mg and sildenafil ranged from 12.5–150 mg. Topical 2% ISDN cream was applied thinly on the wrist three times a day. Patients had to have follow-up data between 2 and 4 months after starting treatment. Patients who lacked follow-up data or used the cream for indications unrelated to RP or DUs were excluded from the study. The following types of patients were recorded: (1) those, who were lost to follow-up after the first prescribing of 2% ISDN cream; (2) those, who used 2% ISDN cream for other indications, such as peripheral vascular disease; and (3) those, who had no outcome (the status of DUs and RP: improvement/stable/worsening). The collected data included gender, age, disease duration, SSc clinical characteristics, co-morbid diseases, current medical treatments, treatment outcomes, and quality of life.

### Drug Formulation

The compounded cream was prepared as an emulsion in a semisolid form for skin application. The active ingredient was isosorbide dinitrate which had been derived from 5 mg sublingual tablets. To prepare 100 g of 2% ISDN cream, 400 tablets of isosorbide dinitrate (5 mg each) were crushed and mixed with 85 g of a hospital cream base (hydrophilic emulsifier base) and were then divided into 10 g packages. The expiration date of the 2% ISDN cream was 6 months from the date of manufacture, and it needed to be stored at 2–8°C.

### Operational Definitions

The diagnosis of SSc was based on the 2013 American College of Rheumatology (ACR)/EULAR Classification Criteria for Systemic Sclerosis. SSc was classified as the limited cutaneous SSc (lcSSc) or diffuse cutaneous SSc (dc-SSc) subset as per LeRoy *et al*. RP is defined when the skin color of the fingers turns white and purplish after contact with the cold and then returns to red after warming. DUs are defined as an area with a visually discernible depth and a loss of continuity of epithelial coverage.^[7]^

The treatment responses of DUs were categorized into 3 levels according to patients’ self-assessment: 1) Improvement referred to the fact that either pain or ulcer size was better than the last visit; 2) Stable referred to the fact that the symptoms of pain and the ulcer size were the same as the last visit; 3) Worsening referred to when either the pain or the ulcer size was worse than the last visit and/or new digital ulcers had developed.

The treatment responses of RP were categorized into 3 levels according to patients’ self-assessment: 1) Improvement referred to the fact that either the frequency or the duration of the RP attack had reduced; 2) Stable referred to when the frequency and duration of the RP attack were the same as the last visit; 3) Worsening referred to when either the frequency or the duration of the RP attack had gotten worse than the last visit.

Quality of Life was evaluated by using the EuroQoL five dimensions (EQ-5D).^[8]^ The time interval of the follow-up was in accordance with the assessments made by the attending physicians.

### Statistical Analyses

Descriptive statistics were used to report the demographic data. The continuous data was displayed as mean ± standard deviation (SD) or medians with interquartile ranges (IQR) as appropriate. The categorical data was displayed as proportions and percentages. The outcomes of the study were reported with the number of patients and in percentages.

## Results

A total of 49 patients from the SSc registry database during the study period were reviewed. Females were more common than males with a ratio of 1.6: 1.0. Most of the patients were dcSSc subset (40 cases, 81.6%). The patients’ demographic data is presented in Table 1.

**Table 1:** The Demographic data

Data	*N* = 49
Age (years), mean ± SD	60 ± 10.1
Female, *n* (%)	30 (61.2)
Comorbid diseases; *n* (%)	19 (38.8)
Hypertension; *n* (%)	7 (14.3)
Diabetes mellitus; *n* (%)	2(4.1)
Dyslipidemia; *n* (%)	10 (20.4)
dcSSc subset; *n* (%)	40 (81.6)
Disease duration (years), mean (IQR)	5.2 ± 4.9
Clinical SSc characteristics	
RP, *n* (%)	42 (85.7)
DUs, *n* (%)	34 (69.4)
Tendon friction rub, *n* (%)	11 (22.4)
Hand deformities, *n* (%)	22 (44.9)
Esophageal involvement, *n* (%)	21 (42.9)
Stomach involvement, *n* (%)	8 (16.3)
Intestinal involvement, *n* (%)	5 (10.2)
Pulmonary fibrosis, *n* (%)	19 (38.8)
Pulmonary arterial hypertension, *n* (%)	5 (10.2)
Renal crisis, *n* (%)	0
Concomitant vasodilators	
CCBs (nifedipine, amlodipine, *n* (%)	36 (73.5)
PDE-5i (sildenafil), *n* (%)	19 (38.8)

SD, standard deviation; SSc, systemic sclerosis; dcSSc, diffuse cutaneous systemic sclerosis; RP, Raynaud’s phenomenon; DU, digital ulcer; CCBs, calcium channel blockers; PDE-5i, phosphodiesterase type 5 inhibitors; EQ-5D, The EuroQoL five dimensions; VAS, visual analogue scale.

Since 2% ISDN cream had been used as an adjuvant therapy for RP and DUs, the clinical response showed that 37 cases (88.1%) had remained stable or had improved in RP, while 5 (11.9%) had worsened. For DUs, 29 cases (85.3%) had remained stable or had improved, while 5 (14.7%) had worsened. Overall, among patients with both RP and DUs, 39 (79.6%) had been stable or had improved, while 10 (20.4%) had worsened.

**Figure 1:**
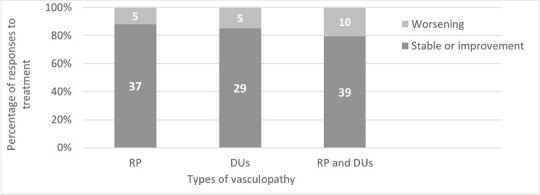
Clinical responses to 2% ISDN categorized by types of vasculopathy. RP; Raynaud’s phenomenon, DUs; Digital ulcer.

The clinical responses to 2% ISDN cream categorized by types of vasculopathy are presented in Figure 1.

Regarding the clinical response to 2% ISDN categorized by the types of vasculopathy, it was found in this study that the Quality of Life by EQ-5D in the dimension of Anxiety/Depression had improved in a statistically significant manner (*P* value = 0.0480). When the DUs had improved and when RP had improved, the Quality of Life by EQ-5D in the dimension of Anxiety/Depression was near significant (*P* value = 0.057). Concerning vasodilators for the treatment of RP and DUs, the study found no statistical significance between CCB and PDE-5i in improving RP and DUs. Some of the patients’ characteristics may have also been important determinants of treatment efficacy. The clinical SSc characteristics of this study, which included comorbid diseases, age, and female gender, were found not to be statistically significant to the improvement of RP and DUs. Also, disease duration did not significantly affect our results. The clinical association with the responses is presented in Table 2.

**Table 2:** The clinical responses to 2% ISDN categorized by types of vasculopathy

		RP			DUs			RP and DUs		
	
Clinical	Stable or improvement *N* = 37	Worsening *N* = 5	*P* value	Stable or improvement *N* = 29	Worsening *N* = 5	*P* value	Stable or improvement *N* = 39	Worsening *N* = 10	*P* value
Age (years); mean±SD	62.2±9.5	54.7±10	0.108	62.0±9.6	62.3±13.4	0.947	61.5±9.8	54.0±9.3	0.108
Female, *n* (%)	22 (59.5)	3 (60.0)	0.999	17 (58.6)	2 (40.0)	0.999	24 (61.5)	6 (60.0)	0.999
Comorbid diseases; *n* (%)									
Hypertension, *n* (%)	5 (13.5)	1 (20.0)	0.551	5 (17.2)	0	0.999	6 (15.4)	1 (10.0)	0.999
Diabetes mellitus, *n* (%)	2 (5.4)	0	0.999	1 (3.45)	0	0.999	2 (5.1)	0	0.999
Dyslipidemia, *n* (%)	8 (21.6)	1 (20.0)	0.999	7 (24.1)	1 (20.0)	0.999	8 (20.5)	2 (20.0)	0.999
dcSSc subset, *n* (%)	29 (78.4)	5 (100.0)	0.564	24 (82.8)	4 (80.0)	0.999	30 (76.9)	10 (100.0)	0.173
Disease duration (years), mean (IQR)	5.1±4.6	6.5±8.1	-	6.6±5.5	3.3±4.2	-	5.7±5.1	2.8±3.3	-
Clinical SSc characteristics									
Tendon friction rub, *n* (%)	6 (16.2)	2 (40.0)	0.237	6 (20.7)	2 (40.0)	0.570	6 (15.4)	4 (40.0)	0.181
Hand deformity, *n* (%)	16 (43.2)	4 (80.0)	0.174	15 (51.7)	4 (80.0)	0.355	17 (43.6)	5 (50.0)	0.737
Esophageal involvement, *n* (%)	17 (46.0)	4 (80.0)	0.343	17 (58.6)	3 (60.0)	0.999	19 (48.7)	3 (30.0)	0.478
Stomach involvement, *n* (%)	6 (16.2)	1 (20.0)	0.999	7 (24.1)	0	0.559	8 (20.5)	0	0.180
Intestinal involvement, *n* (%)	4 (10.8)	1 (20.0)	0.488	4 (11.8)	1(20.0)	0.999	4 (10.3)	1 (10.0)	0.999
Pulmonary fibrosis, *n* (%)	15 (40.5)	2 (40.0)	0.999	11 (32.3)	3 (60.0)	0.627	16 (41.0)	3 (30.0)	0.720
Pulmonary arterial hypertension, *n* (%)	5 (13.51)	0	0.999	3 (10.3)	1 (20.0)	0.488	5 (12.8)	0	0.569
Renal crisis; *n* (%)	0	0	-	0	0	-	0	0	-
Concomitant vasodilators									
CCBs (nifedipine, amlodipine), *n* (%)	27 (73.0)	3 (60.0)	0.613	22 (75.9)	3 (60.0)	0.591	27 (69.2)	9 (90.0)	0.253
PDE-5i (sildenafil), *n* (%)	16 (43.2)	2 (40.0)	0.999	14 (48.3)	2 (40.0)	0.999	17 (43.6)	2 (20.0)	0.278
Quality of life by EQ-5D, improved									
Mobility	31 (83.8)	3 (60.0)	0.237	25 (86.2)	3 (60.0)	0.205	33 (84.6)	8 (80.0)	0.659
Self-care	31 (83.8)	3 (60.0)	0.237	25 (86.2)	3 (60.0)	0.205	33 (84.6)	7 (70.0)	0.364
Usual activities	31 (83.8)	3 (60.0)	0.237	25 (86.2)	3 (60.0)	0.205	33 (84.6)	8 (80.0)	0.659
Pain/Discomfort	29 (78.4)	3 (60.0)	0.577	24 (82.8)	3 (60.0)	0.268	31 (79.5)	8 (80.0)	0.999
Anxiety/Depression	31 (83.8)	2 (40.0)	0.057	25 (86.2)	2 (40.0)	0.048*	33 (84.6)	7 (70.0)	0.364
VAS of health (points), median, (IQR)	27 (73.0)	4 (80.0)	0.999	23 (79.3)	4 (80.0)	0.999	29 (74.4)	9 (90.0)	0.419

*statistically significant. SSc, systemic sclerosis; dcSSc, diffuse cutaneous systemic sclerosis; RP, Raynaud’s phenomenon; DU, digital ulcer; CCBs, calcium channel blockers; PDE-5i, phosphodiesterase type 5 inhibitors; EQ-5D, the EuroQOL five dimensions; VAS, visual analogue scale.

Recently, the updated EULAR recommendations advised oral CCBs as first-line therapy for SSc-related RP, and PDE-5i for patients with SSc with severe RP and/or those who do not respond to CCBs. CCB and PDE-5i have been used for the treatment of RP and DUs. In this study, the results showed no significance between CCB and PDE-5i to improve RP and DUs (*P*-value = 0.421), but the percentage of RP and DUs improvement was found to be higher in those patients who had used CCB (41.0%) instead of PDE-5i (15.4%).

The Clinical response to 2% ISDN categorized by concomitant vasodilators is presented in Table 3.

**Table 3:** The Clinical response to 2% ISDN categorized by concomitant vasodilators

Clinical characteristics	No vasodilator *N* (%)	With CCBs *N* (%)	With PDE-5i *N* (%)	With CCBs and PDE-5i *N* (%)	*P*-values
RP stable or improvement, *n* (%)	5 (13.5)	16 (43.2)	5 (13.5)	11 (29.7)	1.000
DUs stable or improvement, *n* (%)	3 (10.3)	12 (41.4)	4 (13.8)	10 (34.5)	0.910
RP and DUs stable or improvement, *n* (%)	6 (15.4)	16 (41.0)	6 (15.4)	11 (28.2)	0.421

CCBs, calcium channel blockers; PDE-5i, phosphodiesterase-5 inhibitor; RP, Raynaud’s phenomenon; DUs, digital ulcers.

In this study, we found RP and DUs to be stable or to show improvement in those patients, who used CCB at 41%, PDE-5i at 15.4%, and a combination of CCB and PDE-5i at 28.2%. CCB was determined to be more effective than PDE-5i for treating RP and DUs, but this was not significant. We included CCB as the first-line pharmacological treatment. PDE-5i was reserved for more RP, the healing of DUs, or the treatment of pulmonary hypertension. Nineteen patients in this study received PDE-5i, as we found that 5 out of 19 patients had pulmonary hypertension with RP/or DUs and 14 out of 19 patients had severe RP or DUs. As a result, we needed to combine CCB with PDE-5i in the treatment.

## Discussion

We reported the outcomes of open-labeled treatment using topical nitrates in the form of 2% ISDN cream as an adjunctive treatment for vasculopathy in Thai patients with SSc, who had limited use of additional systemic vasodilators. Due to the warm climate in Thailand, the number of patients with vasculopathy requiring further vasodilator treatment was limited. Consequently, only a small number of the patients were included in the study.

According to the demographic data, most of our patients (81.6%) were in the dcSSc subset, in which fibrosis predominated more than vasculopathy when compared to the lcSSc subset. This suggests that dcSSc may also require aggressive treatment to control vasculopathy.

The majority of patients were stable or showed improvement of RP, DUs, and both RP and DUs after treatment with 2% ISDN. The proportion of patients with RP who showed clinical improvement appeared to be higher than those with DUs or those with both RP and DUs. Generally, DUs are indicative of severe vasculopathy and lead to localized tissue ischemia at the digital tip. DUs are often chronic and need long-term recovery.^[9]^ Therefore, a short duration of treatment may not be sufficient to determine whether 2% ISDN is effective in alleviating DUs. Based on our observation, topical nitrates may yield better outcomes in patients with less severe forms of vasculopathy.

CCBs are the most common background vasodilator treatment for RP and DUs in our SSc patients, followed by PDE-5i. In general, extended-release CCBs are preferred as a first-line therapy for RP. If full doses are ineffective, or if DUs develop while on CCBs, then a second vasodilator such as PDE-5i or topical nitroglycerin can be added.^[10]^ Our treatment approach was consistent with the steps outlined in the literature. Although clinical responses to topical 2% ISDN cream had not been significantly different as background treatments for RP and DUs among patients receiving CCBs or PDE-5i or both, the combination of CCBs and/or PDE-5i with 2% ISDN cream as an adjuvant may at least prevent the worsening of RP and/or DUs in SSc patients. A previous systematic review and meta-analysis of the effects of topical nitrates showed significant efficacy in the treatment of both primary and secondary RP as an adjunctive therapy.^[6]^ Given the primary mechanism of action of topical nitrates in improving digital blood flow, there is, however, no data on their use as an adjunctive treatment for DUs. A randomized controlled trial is, therefore, recommended to determine the efficacy of topical nitrates for DUs.

In this study, we also collected data on atherosclerosis conditions, such as high blood pressure, diabetes, and high levels of cholesterol in the blood. RP can occur in individuals with atherosclerosis and is referred to as secondary RP. A previous study found that the prevalence of high blood pressure was at 14.3%, diabetes was at 4.1%, and high cholesterol levels were at 20.4%. All of which could contribute to worsening RP. A meta-analysis showed that SSc patients have a higher prevalence of coronary atherosclerosis, peripheral vascular disease, and cerebrovascular calcification compared to healthy controls.^[11]^ Since no atherosclerosis was confirmed in our patients, we still question whether peripheral vascular disease may contribute to the difficulty in treating RP and/or DUs. We suggest that patients with coexisting atherosclerosis conditions should improve their diet, exercise regularly, and maintain control of their blood pressure, cholesterol level, and diabetes to aid in the improvement of vasculopathy.

Topical nitrates may improve QoL in patients with vasculopa-thy. Our study included the EQ-5D as an outcome measure for the treatment of SSc. This study found that as an adju-vant, 2% ISDN cream had shown significant improvement to the QoL in terms of anxiety among patients with DUs and had tended to improve anxiety among those with RP. As we know, RP and DUs can negatively impact QoL, due to local pain, and difficulty performing daily activities, all of which can lead to anxiety.^[12]^ Topical nitrates may help to reduce anxiety and improve the patient’s overall QoL. Due to the small population of the study, there was a statistical limitation to confirm the efectiveness of 2% ISDN on the other domains of QoL. Further study in a larger population is, therefore, suggested. The safety of topical nitrates is a concern when added to va-sodilator therapy. The combination of 2% ISDN with CCBs and/or PDE-5i may cause adverse effects, such as headaches and hypotension. However, these adverse effects were assessed, and there were no reports of patients experiencing these side effects. Therefore, 2% ISDN might be a viable treatment option for those, who are intolerant to CCBs or PDE-5i.

This study had some limitations. Firstly, the sample size was small, and the study was conducted at a single center, which may limit the generalizability of the findings. A larger cohort or randomized controlled trials are needed to confirm the results. Secondly, the study did not collect detailed information regarding the frequency and duration of RP attacks, nor did it measure digital ulcer size. Treatment responses were based on patient-reported outcomes categorized into improvement, stability, or worsening. Thirdly, the follow-up period ranged from 2 to 4 months, but the effects of discontinuing the cream were not evaluated as this study focused on continuous use. Additionally, patients who did not have follow-up data were excluded from the analysis, which may have introduced bias as the outcomes for these patients remain unknown. The interval between the initiation of 2% ISDN cream and background therapy with CCBs or PDE-5i was also not systematically recorded. Finally, the study did not include inflammatory markers or specific serological results for SSc. Consequently, the influence of inflammation or serology type on clinical responses could not be determined. In contrast, the strengths of our study include being the first to apply a topical vasodi-lator as adjunctive therapy for vasculopathy in SSc and incorporating QoL as a study outcome, which allowed us to evaluate the medication’s impact on improving patients’ QoL

## Conclusion

Topical 2% ISDN cream may help to reduce anxiety and improve overall QoL. No adverse reactions were reported. It may be used as an adjunctive treatment alongside CCBs and/or PDE-5i in SSc patients with RP and/or DUs, who are unable to tolerate vasodilators. However, a well-designed randomized controlled trial is recommended to confirm the efficacy of 2% ISDN.
